# Comparative Physiological and Transcriptomic Characterisation of Two Japonica Rice Cultivars Under Low Nitrogen Stress

**DOI:** 10.3390/plants14243836

**Published:** 2025-12-16

**Authors:** Yu Zou, Yi Ren, Shuxin Jiang, Xinchun Zhan, Peijiang Zhang, Shaojie Song, Ending Xu

**Affiliations:** 1 Anhui Province Key Laboratory of Rice Germplasm Innovation and Molecular Improvement, Rice Research Institute, Anhui Academy of Agricultural Sciences, Hefei 230031, China; 2 College of Agricultural, Anhui Science and Technology University, Chuzhou 233100, China

**Keywords:** rice, transcriptome, cultivars, root physiology, nitrogen use efficiency

## Abstract

Nitrogen (N) is an essential nutrient for the growth and development of rice. However, excessive N fertiliser application and low N Use Efficiency (NUE) have led to serious environmental problems and threatened agricultural sustainability. In this study, we compared the physiological and transcriptomic profiles of roots of two cultivars exposed to normal nitrogen (NN) and low nitrogen (LN). The results showed that the LN treatment suppressed root growth and severely affected enzymatic activities in the roots of both rice cultivars compared to the NN treatment. Moreover, HJ753 exhibited significantly higher activities of NITRATE REDUCTASE (NR) and GLUTAMINE SYNTHETASE (GS) in its roots than DJ8 under both LN and NN conditions. Transcriptomic analysis identified 23,205 genes across all samples, with more than 5000 differentially expressed genes (DEGs) detected in response to LN stress in both cultivars. The KEGG analysis revealed that the DEGs were primarily involved in DNA replication, tryptophan metabolism, phenylpropanoid biosynthesis, plant hormone signal transduction, and N metabolism. Under LN stress, most genes associated with tryptophan metabolism and phenylpropanoid biosynthesis pathways remained stable or were upregulated in both cultivars. In contrast, genes related to auxin signalling transduction, N metabolism, and N utilisation exhibited significant genotype-specific expression patterns between HJ753 and DJ8. In conclusion, this study elucidated the genotypic differences in root development and N response mechanisms under LN stress at the molecular level, providing new insights into the regulatory mechanisms of N efficiency that may be used to develop and support the breeding of N-efficient rice cultivars.

## 1. Introduction

Nitrogen (N) is a crucial nutrient element that significantly influences plant growth, development, and yield and also plays vital roles in various cellular metabolic processes [1]. Hence, in pursuit of higher yields, nitrogen fertilisers are often applied excessively in agricultural production. However, the average N uptake efficiency by crops is generally low, ranging only from 30 to 40% [2]. This pattern of high input coupled with low efficiency increases production costs and leads to water eutrophication and soil compaction, which severely constrain the sustainability of rice production systems [3,4]. Therefore, reducing the application of N fertiliser while enhancing its use efficiency is critical in achieving sustainability in modern agriculture.

The morphological and physiological characteristics of the root system are key determinants of the absorption and utilisation efficiency of N in rice, with N-efficient rice cultivars typically containing roots with greater biomass, deeper distribution, longer length, and higher density [5]. Rice can also activate a root “foraging response” by modifying the root architecture in response to changes in the availability of soil nitrogen to enhance its perception and uptake in the rhizosphere [6]. Further, the acquisition of N primarily depends on the activity of NITRATE TRANSPORTERS (NRTs) and AMMONIUM TRANSPORTERS (AMTs), which mediate its uptake and also play regulatory roles in root morphological development, and their high expression levels are often correlated with improved N acquisition and utilisation efficiency under N-limited environments. For instance, *OsNRT2.4* simultaneously promotes N uptake and lateral root growth, while low N impairs *OsAMT1.3*, significantly reducing the number and length of lateral roots [7,8]. After acquisition, N is assimilated into organic compounds through a series of physiological and biochemical processes [9]. For example, the activity of NR, GS, and GLUTAMATE SYNTHASE (GOGAT) enzymes in N assimilation serves as an important physiological indicator of root metabolic capacity. In N-efficient rice cultivars, these enzymes generally demonstrate higher activity, ensuring efficient assimilation and utilisation of N [10,11].

The process of absorption and utilisation of N in plants is highly complex, involving multiple activities, including uptake, assimilation, metabolism, and regulation, which cannot be fully explained by a limited number of genes [12]. Recently, transcriptome sequencing has become a crucial tool for investigating the mechanisms of low-N tolerance in rice, enabling systematic dissection of the genetic basis underlying complex traits under N limitation. For instance, studies have identified 1158 and 492 N-starvation-responsive DGEs in the leaf sheath and roots of rice, respectively [13], while comparative transcriptomic analysis of young panicles from japonica rice exposed to high and low N revealed 4309 DEGs and predicted three candidate N utilisation genes [14]. Furthermore, cross-species comparison between rice and Arabidopsis uncovered 73 orthologous gene clusters responsive to N starvation, suggesting conserved mechanisms of N stress response across plants [15]. Under low-N stress, roots exhibit extensive differential gene expression, with most of the DEGs broadly participating in carbon and N metabolic processes, such as glycolysis, oxidative phosphorylation, starch and sucrose metabolism, N transport and assimilation, and amino acid metabolism [16]. RNA-Seq analysis in maize has also indicated that N-efficient traits under low-N conditions are closely associated with high expression of genes related to antioxidant activity and respiration in the root system. Additionally, different crop varieties exhibit significant variations in their physiological responses to LN stress. Compared to LN-sensitive varieties, those with LN tolerance generally demonstrate superior adaptive capacity [17]. Therefore, transcriptomic profiling and functional enrichment analysis offer a powerful approach to systematically uncover core genes and key metabolic pathways involved in response to changes in N availability or its stress in different rice cultivars.

Although it is well established that nitrogen significantly regulates rice root system architecture, how LN conditions drive N utilisation in roots by reprogramming the global gene expression profile to precisely orchestrate metabolic pathways needs further elucidation. In this study, the two cultivars were subjected to LN treatment at the seedling stage and used to systematically analyse differences in their root morphology, physiological activity, and transcriptomic responses. The integrated approach revealed genotype-specific adaptations in root system architecture and identified key genes and metabolic pathways involved in the response or adaptation of rice to low N and also offers a theoretical foundation for breeding N-efficient rice cultivars.

## 2. Results

### 2.1. Effect of LN on the Root System of Rice Seedlings

**Figure 1:**
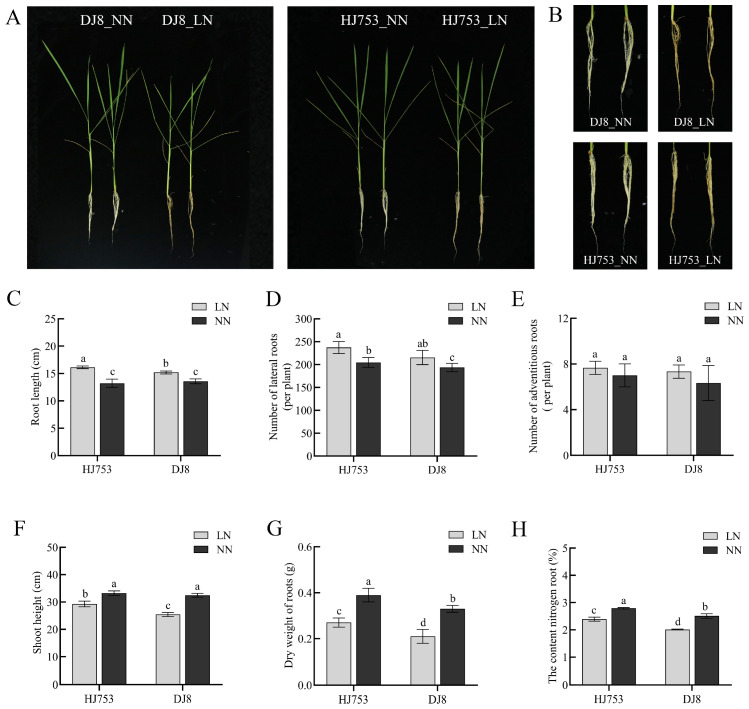
Morpho-physiological analyses of HJ753 and DJ8 seedlings: (**A**) 10-day-old seedlings of rice cultivars HJ753 and DJ8 were grown for 10 days under normal-nitrogen (NN) or low-nitrogen (LN) hydroponic conditions (*n* = 192 plants per cultivar). (**B**) Image of magnified root system. (**C**) Root length. (**D**) Number of lateral roots. (**E**) Number of adventitious roots. (**F**) Shoot height. (**G**) Root dry weight. (**H**) Nitrogen content in roots. Data represent the mean ± SD (*n* = 3). Different letters above bars indicate significant differences (one-way ANOVA followed by Tukey’s test, *p* < 0.05). Scale bars = 8 cm in (**A**), 4 cm in (**B**).

Compared with the NN condition, LN stress significantly inhibited the growth (Figure 1A,B). HJ753 and DJ8 exhibited a notable increase in root length and lateral root number under LN treatment, although the number of adventitious roots showed no significant difference (Figure 1C–E). LN increased the root length and lateral root number by 41.6% and 15.9% in HJ753 and by 18.4% and 11.2% in DJ8, respectively (Figure 1C,D). Additionally, low-nitrogen stress significantly reduced shoot height, root dry weight, and root nitrogen content. Specifically, shoot height decreased by 11.8% and 21.7% in HJ753 and DJ8, respectively. Reductions of 30.8% and 36.4% were observed for root dry weight, while root nitrogen content declined by 14.0% and 19.9% in HJ753 and DJ8, respectively (Figure 1F–H). Quantification and analysis of variance data are provided in Table S1.

### 2.2. Analysis of Root Enzyme Activities

**Figure 2:**
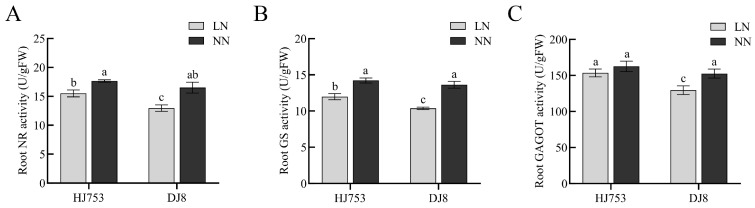
Enzyme activity assays in roots of rice cultivars HJ753 and DJ8: 10-day-old seedlings of rice cultivars HJ753 and DJ8 were grown for 10 days under normal-nitrogen (NN) or low-nitrogen (LN) hydroponic conditions. Enzyme activities in roots were measured for NITRATE REDUCTASE (NR) (**A**), GLUTAMINE SYNTHETASE (GS) (**B**), and GLUTAMATE SYNTHASE (GOGAT) (**C**). Data represent the mean ± SD (*n* = 5). Different letters above bars indicate significant differences (one-way ANOVA followed by Tukey’s test, *p* < 0.05).

Significant differences in NR, GS, and GOGAT activities emerged both between and within the two rice cultivars under LN and NN conditions. HJ753 consistently exhibited higher activities of all three enzymes compared to DJ8 (Figure 2A–C). Under LN stress, the activities of NR and GS in both cultivars decreased relative to NN conditions. Specifically, HJ753 showed reductions of 11.6% in NR activity and 15.8% in GS activity, while DJ8 exhibited a more pronounced decrease, with reductions of 21.5% in NR and 23.9% in GS (Figure 2A,B). In contrast, GOGAT activity in HJ753 remained largely unchanged under LN treatment, whereas a significant decline was observed in DJ8 (Figure 2C). Quantification and analysis of variance data are provided in Table S1.

### 2.3. RNA Sequencing Analysis

In this study, RNA extraction and transcriptome sequencing were performed on four treatment groups, including HJ753_LN, HJ753_NN, DJ8_LN, and DJ8_NN, each in triplicate. After quality control, a total of 560 million high-quality library reads were obtained, amounting to 84.01 GB of data (Table S2). The effective data volume per sample ranged from 5.84 to 7.73 GB, while the Q20 and Q30 base scores were between 98.27 and 99.29% and 95.08 and 97.36%, respectively, with an average GC content of 49.80% (Table S3). The proportion of clean paired-end reads across all samples ranged from 74.71 to 83.06% (Table S3). On the other hand, the alignment of the generated reads with the reference genome showed an overall mapping efficiency of 80.70–89.33%, with uniquely mapped reads accounting for 78.42–87.02%, while multiply mapped reads comprised 2.04–2.45%. All the samples exhibited a Q30 value above 95.08%, with a GC content ranging from 48.88 to 50.37% (Table S3). These results indicated that the sequencing data were of high quality and suitable for subsequent bioinformatic analyses.

Based on FPKM values, we performed principal component analysis (PCA) and inter-sample Pearson correlation analysis based on FPKM values to identify and exclude potential outliers. The PCA results demonstrated clear segregation of samples within each experimental group, indicating strong intra-group reproducibility. The first and second principal components explained 37.09 and 24.75% of the total variance, respectively (Figure S1A). Further analysis revealed that gene expression differences between cultivars were greater than those introduced by LN treatment within each variety. A correlation heatmap showed that all pairwise correlation coefficients between samples were above 0.87, supporting high biological reproducibility across treatments (Figure S1B). In summary, the observed gene expression variations were primarily attributed to the interaction between variety and N level, with variety being the dominant factor and N treatment playing a secondary role.

### 2.4. Analysis of DEGs Within and Between Cultivars Under LN or NN Conditions

**Figure 3:**
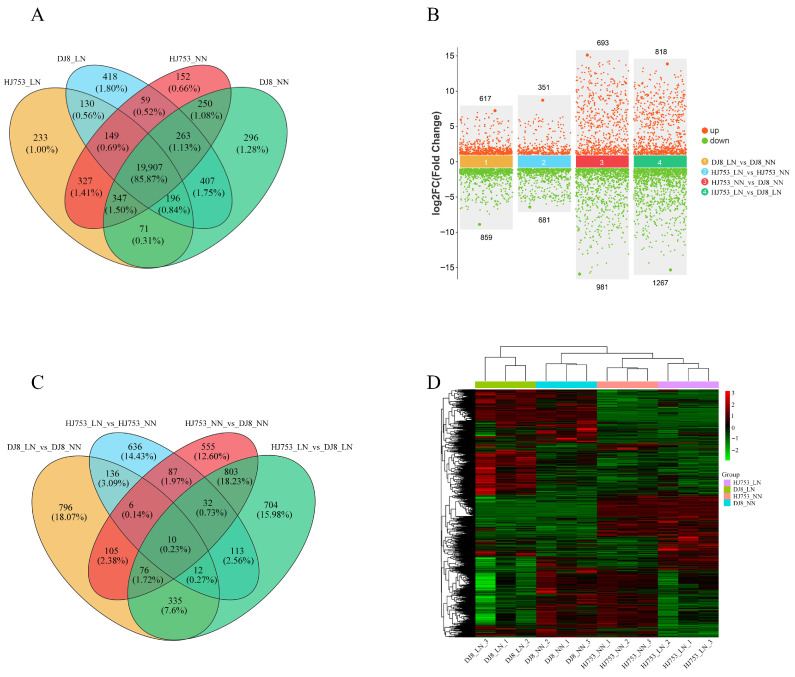
Statistical and cluster analysis of differentially expressed genes: 10-day-old seedlings of rice cultivars HJ753 and DJ8 were grown for 10 days under normal-nitrogen (NN) or low-nitrogen (LN) hydroponic conditions. Root RNA was extracted for transcriptome sequencing. (**A**) Venn diagram of co-expressed genes across comparisons. (**B**) Identification of differentially expressed genes (DEGs) in within- and between-variety comparisons; red dots represent up-regulated genes and green dots represent down-regulated genes. (**C**) Venn diagram illustrating overlaps of DEG sets among different comparison groups. (**D**) Heatmap of normalised gene expression levels across samples. Each column represents one sample, and each row corresponds to one gene. Colours indicate normalised expression values, with red representing higher expression and green indicating lower expression.

Based on the FPKM values, we further analysed the gene expression profiles of HJ753 and DJ8 cultivars under different N treatments to elucidate transcriptomic changes in response to N within each genotype. A total of 23,205 genes were detected across all samples, with 21,357, 21,454, 21,193, and 21,737 genes in the HJ753_LN, HJ753_NN, DJ8_LN, and DJ8_NN treatment groups, respectively (Figure 3A). Of the 23,205 genes, 19,907 (85.87%) were commonly detected in all groups, indicating a stable transcriptional background across all the cultivars and treatments.

We identified 4406 DEGs across the four comparison groups generated, including DJ8_LN_vs_DJ8_NN, HJ753_LN_vs_HJ753_NN, HJ753_NN_vs_DJ8_NN and HJ753_LN_vs_DJ8_LN. In the DJ8_LN_vs_DJ8_NN comparison, 617 DEGs were up-regulated and 859 were down-regulated, while in the HJ753_LN_vs_HJ753_NN comparison, 351 DEGs were up-regulated and 681 were down-regulated (Figure 3B). For the HJ753_NN_vs_DJ8_NN comparison, 693 genes were up-regulated and 981 were down-regulated, while in the HJ753_LN_vs_DJ8_LN comparison, 818 transcripts were up-regulated and 1267 were down-regulated (Figure 3B). Additionally, there are 10 differentially expressed genes in all groups (Figure 3C). Clustering analysis also revealed that biological replicates within each variety grouped closely, demonstrating high reproducibility (Figure 3D). These results indicate that the transcriptional responses to LN stress markedly differ between HJ753 and DJ8 cultivars, highlighting genotype-specific adaptive mechanisms.

### 2.5. The GO Analysis of the DEGs Within and Between Cultivars Under LN or NN Conditions

**Figure 4:**
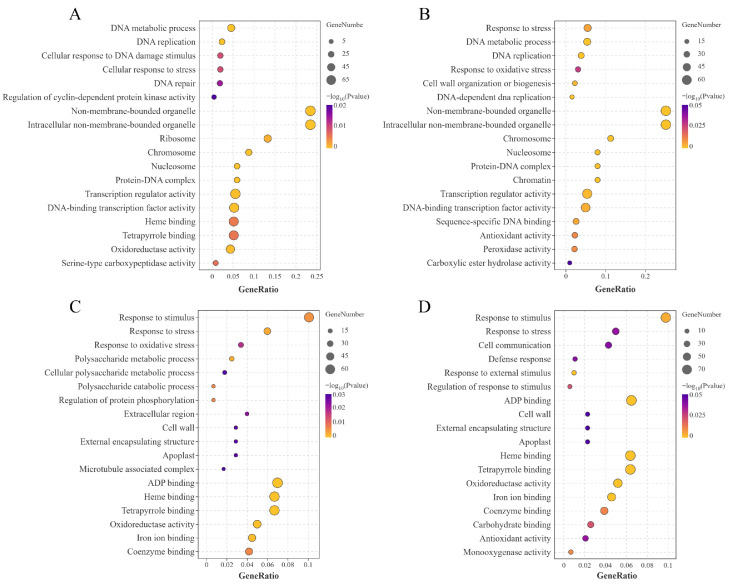
Diversion of enriched GO pathways between the two rice cultivars. Significantly enriched GO pathways identified from DEGs in the following comparisons: (**A**) DJ8_LN vs. DJ8_NN, (**B**) HJ753_LN vs. HJ753_NN, (**C**) DJ8_NN vs. HJ753_NN, and (**D**) DJ8_LN vs. HJ753_LN.

To further elucidate the biological functions of DEGs in HJ753 and DJ8 cultivars under LN stress, GO enrichment analysis, which categorises gene functions into biological process, cellular component, and molecular function, was performed using the GO database. The results revealed that in within-variety comparisons (HJ753_LN vs. HJ753_NN and DJ8_LN vs. DJ8_NN), DEGs were significantly enriched in GO terms associated with transcriptional regulation and oxidative stress response, including DNA metabolic process, response to stress, nucleosome, transcription regulator activity and oxidoreductase activity (Figure 4A,B). In between-variety comparisons (HJ753_NN vs. DJ8_NN and HJ753_LN vs. DJ8_LN), DEGs were notably linked to response to stimulus, response to stress, cell wall, as well as molecular functions such as heme binding and tetrapyrrole binding (Figure 4C,D). These findings suggest that different genotypes may activate distinct biological pathways and molecular functions to adapt to LN conditions.

### 2.6. KEGG Analysis of DEGs Within and Between Cultivars Under LN or NN Conditions

**Figure 5:**
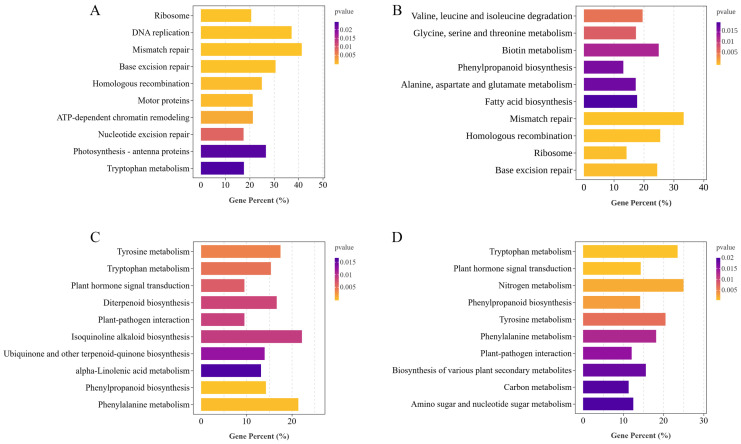
Diversion of enriched KEGG pathways between the two rice cultivars. Significantly enriched KEGG pathways identified from DEGs in the following comparisons: (**A**) DJ8_LN vs. DJ8_NN, (**B**) HJ753_LN vs. HJ753_NN, (**C**) DJ8_NN vs. HJ753_NN, and (**D**) DJ8_LN vs. HJ753_LN.

To further investigate the biological pathways involved in the response to LN stress in HJ753 and DJ8 rice cultivars, KEGG analysis was performed on the identified DEGs (Figure 5). Under LN treatment, DEGs in DJ8 were significantly enriched in KEGG pathways, such as ribosome, photosynthesis-antenna proteins, DNA replication, and tryptophan metabolism (Figure 5A). In contrast, the DEGs in variety HJ753 were primarily enriched in amino acid metabolism-related pathways, such as valine, leucine, and isoleucine degradation, glycine, serine, and threonine metabolism, alanine, aspartate and glutamate metabolism, and phenylpropanoid biosynthesis (Figure 5B). The DEGs involved between HJ753 and DJ8 under NN and LN conditions were significantly enriched in tryptophan metabolism, diterpenoid biosynthesis, plant hormone signal transduction, and N metabolism pathways (Figure 5C,D). These findings suggest that HJ753 and DJ8 cultivars may employ distinct metabolic strategies in response to N deficiency and modulate different biological pathways to adapt to LN conditions.

### 2.7. Expression Analysis of Gene Family Members in Metabolic Pathways

**Figure 6:**
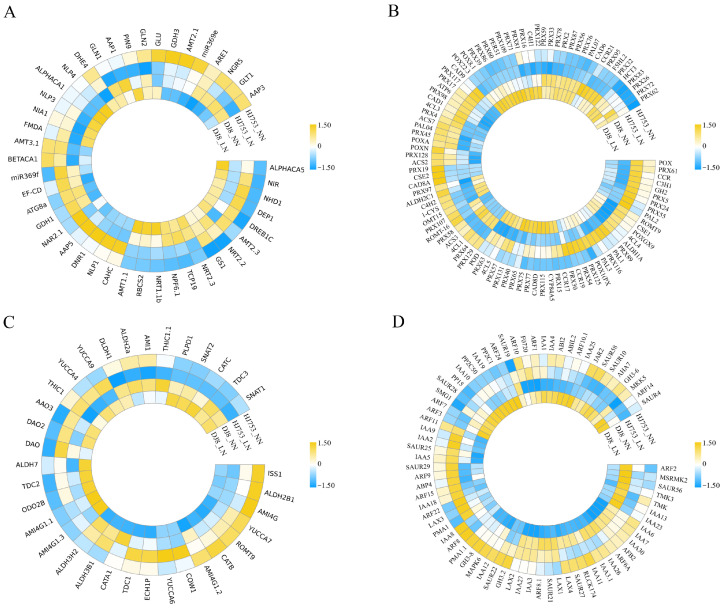
Heatmap of transcriptomic changes in metabolism-related genes. Expression profiles are shown for genes involved in (**A**) nitrogen metabolism and utilisation pathway; (**B**) phenylpropanoid biosynthesis pathway; (**C**) tryptophan metabolism pathway; (**D**) auxin signal transduction pathway. The heatmap displays normalised gene expression levels across samples. Each column represents an individual gene. Colours indicate normalised expression values, with yellow denoting higher expression and blue indicating lower expression in the corresponding sample.

To further investigate the metabolic regulatory networks mediated by DEGs in the HJ753 and DJ8 rice cultivars under LN stress, the current study focused on four significantly enriched pathways, tryptophan metabolism, phenylpropanoid biosynthesis, N metabolism, and the auxin signalling pathway, within plant hormone signal transduction (Figure 6), which are also closely associated with plant response to N deficiency. We also examined the expression levels of previously reported N utilisation-related genes [18]. Most genes within the N metabolism and N utilisation-related pathways remained unchanged or were up-regulated under LN conditions, with only a limited number being down-regulated (Figure 6A). Specifically, genes involved in root ammonium absorption, including *AMT2*, *LEAF FERREDOXIN-NITRITE REDUCTASE* (*NIR)*, and *DENSE AND ERECT PANICLE 1* (*DEP1*) and *NIN-LIKE PROTEIN 1/3* (*NLP1/3*), were up-regulated in both DJ8 and HJ753 under LN stress (Figure 6A). On the other hand, *miR369f*, *ATG8a*, and *GDH1* genes were down-regulated in DJ8 but up-regulated in HJ753 (Figure 6A). These results indicate distinct molecular mechanisms between DJ8 and HJ753 in response to LN stress.

In the phenylpropanoid biosynthesis pathway, the majority of genes in both DJ8 and HJ753 cultivars were not significantly affected by LN treatment, with no marked changes in their transcriptional levels (Figure 6B). However, several genes, such as *PEROXIDASE 115* (*PRX115*), *PRX131*, and *CINNAMOYL-COA REDUCTASE 17* (*CCR17*), were up-regulated in both cultivars, while *PRX107* and *4-COUMARATE:COA LIGASE 1* (*4CL1*) were commonly down-regulated (Figure 6B).

In the tryptophan metabolism pathway, most genes were either up-regulated or unchanged under LN conditions in both cultivars, with only a limited number being down-regulated (Figure 6C). Commonly up-regulated genes in HJ753 and DJ8 included *DIOXYGENASE FOR AUXIN OXIDATION 2* (*DAO2*), *TRYPTOPHAN DECARBOXYLASE 2* (*TDC2*), *FLAVIN-CONTAINING MONOOXYGENASE -like gene 4* (*YUCCA4*), and *YUCCA9*. Interestingly, *YUCCA 6* was specifically up-regulated only in HJ753 (Figure 6C). Within the core Auxin signal transduction pathway, three key gene families, *INDOLE ACETIC ACID* (*IAA*), *SMALL AUXIN-UP RNA* (*SAUR*), and *AUXIN RESPONSE FACTORS* (*ARF*), exhibited significant transcriptional differences under LN stress (Figure 6D). Most genes showed altered expression levels in either DJ8 or HJ753, with distinct up- or down-regulation patterns between the two cultivars, indicating genotype-specific regulatory mechanisms in response to N deficiency.

To validate the gene expression patterns, 12 genes were selected from four metabolic pathways for qRT-PCR analysis (Figure S2). A comparison between the qRT-PCR results and RNA-seq data revealed highly consistent expression patterns of the selected genes, thereby confirming the reliability of the RNA-seq data.

## 3. Discussion

### 3.1. Root and Physiological Changes in Rice Seedlings Under LN Conditions

As the primary organ for nutrient acquisition, the root system plays a central role in the adaptation of plants to nutrient deficiency [1]. For instance, under LN stress, the roots morphologically and physiologically undergo adaptive changes through the N foraging response, which constitutes an important functional basis for efficient utilisation of N in plants [6]. However, the patterns of root morphological changes under LN stress may vary in different crop species and among genotypes within the same species. In the current study, after exposing rice seedlings to LN for 10 days, both HJ753 and DJ8 exhibited significantly increased root length and lateral root number compared to the controls (Figure 1B–D), though the number of adventitious roots remained unchanged. Previous studies have demonstrated that both short-term nitrogen starvation and localised nitrogen supply can induce root growth and lateral root formation in rice, thereby enhancing nitrogen uptake [19]. This adaptive response is primarily attributed to alterations in internal hormone homeostasis and a reallocation of photosynthetic carbon resources to the roots under nitrogen deficiency. These changes subsequently activate a root “foraging program”, ultimately optimising the root system architecture to cope with the N-deprived environment [12].

NR, GS, and GOGAT are key enzymes in plant N metabolism, playing crucial roles in the assimilation of inorganic N [20]. The NR serves as the rate-limiting enzyme in N assimilation, catalysing the reduction of NO_3_^−^ to NH_4_^+^, which is subsequently converted into glutamine by GS, and further transformed into glutamate via GOGAT catalysis. These products serve as essential N donors and carbon skeletons for the synthesis of amino acids, proteins, nucleic acids, chlorophyll, and other N-containing compounds [21]. In the current study, HJ753 exhibited significantly higher activities of NR and GS in its roots than DJ8 under both LN and NN conditions, indicating its superior capacity for N uptake and transformation (Figure 2A,B). Additionally, we observed a declining trend in GOGAT activity in both rice cultivars under LN stress (Figure 2C). This decline may be attributed to the preferential allocation of limited ATP and NAD(P)H reserves to maintain essential survival and respiratory metabolism rather than support growth-related N assimilation processes [22,23]. These results collectively demonstrate the discrete physiological responses involved in the root systems of HJ753 and DJ8 under LN conditions.

### 3.2. The Expression of Genes Involved in Biological Metabolism Pathways Under LN Conditions

The molecular regulatory network established in the roots of rice under LN stress is highly sophisticated and complex. This system integrates a multi-level signal transduction to coordinate a continuous biological process ranging from N signal perception to the remodelling of root architecture plasticity, ultimately enhancing N acquisition capacity [12]. Ammonium N (NH_4_^+^-N) and nitrate N (NO_3_^−^-N) are the two primary inorganic N sources absorbed and utilised by plants, and their transmembrane transport depends on AMTs and NRTs, respectively [24,25]. In this study, under LN conditions, the expression of the high-affinity nitrate transporter gene *OsNRT2.2* was significantly up-regulated in the roots of the HJ753 rice variety, unlike in DJ8, where no change was observed (Figure 6A). Previous studies have indicated that the expression of *OsNRT2.2* is induced by nitrate but suppressed by NH_4_^+^ [26]. In this study, the nitrate transport accessory protein gene *OsNAR2.1* was up-regulated in HJ753 under LN stress, while an insignificant response was detected in DJ8 (Figure 6A). *OsNAR2.1* can interact with *OsNRT2.2* to form a functional complex and synergistically enhance NO_3_^−^ uptake across a broad range of nitrate concentrations. Furthermore, insignificant changes were observed in the expression of other *OsNRT2* family genes in either variety (Figure 6A), suggesting that different NRTs may fulfil distinct physiological functions, and their regulatory mechanisms are likely influenced by N form and nutritional status [27].

We also found a significant up-regulation of the ammonium transporter gene, *AMT2.3*, in the roots of both rice cultivars under LN stress (Figure 6A), consistent with previous reports, confirming that the expression of *AMT2.3* is regulated by N levels [28]. Key transcription factors in the N signalling pathway, such as members of the *NLP* family, coordinate to regulate multiple genes involved in N uptake and assimilation, thereby modulating the utilisation of nitrate and ammonium nitrogen [29]. In this study, *NLP1* and *NLP4* exhibited distinct transcriptional patterns between DJ8 and HJ753 under LN stress (Figure 6A), suggesting their potential roles in mediating the divergence in N use efficiency between the two cultivars.

Inorganic N sources absorbed by rice must be assimilated into organic N compounds before being integrated and utilised. In this process, GS catalyses the ATP-dependent conversion of NH_4_^+^ and glutamate into glutamine, representing a critical step in N assimilation. Previous studies have shown that *OsGS1* plays an essential role in the primary assimilation of NH_4_^+^-N following its uptake by rice roots [30]. In this study, we further revealed that the expression response of *OsGS1* to LN stress is genotype-dependent. For example, the expression of *OsGS1* was not induced in the DJ8 variety under LN conditions, but was significantly up-regulated in the HJ753 variety (Figure 6A). Several other key functional genes involved in N metabolism, including *GLU* and *miR369f,* also exhibited notable transcriptional differences between the two cultivars under LN treatment [31,32]. Collectively, the differential expression patterns of these genes are likely to regulate phenotypic variations in N uptake, assimilation, and remobilisation efficiency between DJ8 and HJ753, ultimately contributing to their divergence in N use efficiency.

Low-N stress typically induces substantial accumulation of secondary metabolites in plants, particularly phenylpropanoids. As one of the largest classes of natural products in plants, phenylpropanoids consist of important compounds, such as flavonoids, anthocyanins, and lignins, which play versatile roles in photosynthesis, nutrient uptake, maintenance of structural integrity, and responses to both biotic and abiotic stresses [33]. In this study, KEGG analysis revealed that DEGs responsive to LN stress were significantly enriched in the phenylpropanoid biosynthesis pathway (Figure 5). The initial and committed step of this pathway is catalysed by *PHENYLALANINE AMMONIA-LYASE* (*PAL*), which deaminates phenylalanine to form cinnamic acid while releasing ammonium ions [34]. Under LN conditions, we observed significant up-regulation of *PAL1/2/3* in HJ753 and *PAL1/3* in DJ8 (Figure 6B), which suggests that the deficiency of N triggers a metabolic shift in plants, diverting resources from protein synthesis towards the production of phenolic compounds [35]. The ammonium ions released via the PAL-catalysed reaction can be reassimilated and recycled through the GS/GOGAT cycle, forming a N retrieval mechanism that helps mitigate its starvation by replenishing amino acid biosynthesis [36]. Furthermore, analysis of gene expression differences showed that most genes involved in phenylpropanoid metabolism were either up-regulated or stably expressed in both DJ8 and HJ753 under low-N treatment (Figure 6B). For instance, key enzymes involved in lignin biosynthesis, including *CCR17*, *PRX115*, and *CINNAMYL ALCOHOL DEHYDROGENASE* (*CAD8D*), exhibited significant up-regulation in both cultivars under LN conditions (Figure 6B). This indicates that under the deficiency of N, protein synthesis is constrained, causing plants to redirect relatively abundant carbon skeletons toward the synthesis of N-free carbon-based compounds. This process not only enhances cell wall lignification and physical defence but also optimises carbon reallocation under stress conditions [37].

KEGG analysis further revealed significant alterations in multiple amino acid metabolic pathways under LN stress, with the tryptophan metabolism pathway being particularly prominent (Figure 5). Tryptophan serves as an essential amino acid for protein synthesis, and also plays a pivotal role in plant LN tolerance [38]. Studies have demonstrated that exogenous tryptophan application can enhance crop adaptation to LN conditions by modulating root architecture and strengthening N stress responses. For instance, exogenous tryptophan treatment significantly elongated sorghum roots and improved LN tolerance, which is primarily attributed to the role of tryptophan as a biosynthetic precursor of IAA, an important signalling molecule [39]. IAA integrates N signalling pathways to regulate root development, thereby modulating N uptake efficiency and establishing an adaptive response cycle [40].

In the auxin biosynthesis pathway, enzymes of the YUCCA family catalyse the rate-limiting step [41]. Our current study revealed that genes encoding these enzymes, specifically *YUCCA4* and *YUCCA9*, were up-regulated under LN stress in both DJ8 and HJ753 cultivars, while *YUCCA6* was only induced in HJ753 (Figure 6C). Additionally, the expression of the *DAO2* gene, which is involved in auxin oxidative catabolism, was increased in both cultivars (Figure 6C). This distinct expression pattern likely reflects a mechanism for fine-tuning auxin homeostasis under N deficiency [42]. Therefore, plants balance biosynthesis and degradation processes to avoid the excessive accumulation of IAA, which can lead to undesirable shoot elongation. Instead, resources are preferentially allocated to root development and the maintenance of essential metabolic processes, ultimately enhancing survival and adaptive capacity in environments with limited N [43]. Consistently, under LN conditions, most members of the core auxin signalling pathway components, including *IAA*, *SAUR*, and *ARF* gene families, exhibited significant transcriptomic changes in both DJ8 and HJ753, though with distinct regulatory patterns between the two cultivars (Figure 6D). The *SAUR* family genes, which are rapidly induced by auxin, promote root cell elongation and directly expand the root absorption surface area, thereby improving nutrient foraging ability in environments deficient in N [44]. This process is regulated by ARF, which recognises and binds specifically to auxin response elements in the promoter regions of downstream genes to activate or repress their transcription [45]. Concurrently, IAA proteins act as negative regulators of auxin signalling by inhibiting the transcriptional activity of *ARFs*, thereby forming a feedback loop [46]. The observed transcriptional differences in these core components between the two rice genotypes may constitute an important intrinsic factor subsequently contributing to divergent root system architecture and N acquisition efficiency under LN stress.

## 4. Materials and Methods

### 4.1. Plant Materials and Growth Conditions

**Figure 7:**
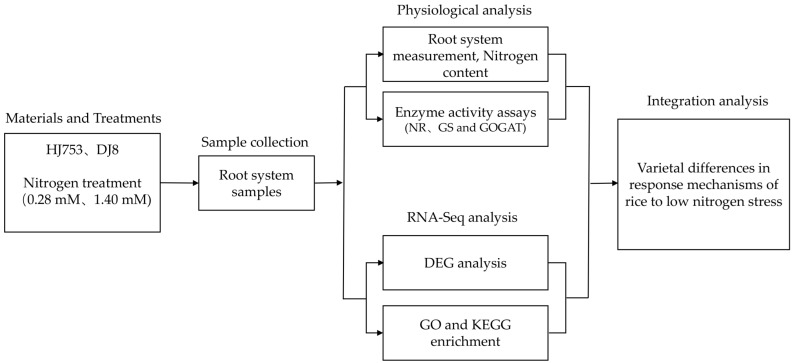
The flowchart of data collection and method implementation in this study.

This study was conducted in 2024 in controlled growth chambers at the experimental station of the Rice Research Institute, Anhui Academy of Agricultural Sciences (RRI-AAAS), using HJ753 and DJ8 japonica rice varieties (*Oryza sativa* L), provided by the institute. The rice seeds were surface-sterilised using 3% sodium hypochlorite solution for 30 min with gentle agitation every 5 min to ensure uniform disinfection, followed by thorough rinsing with distilled water. The seeds were then soaked in distilled water for 48 h and then transferred to a humid environment for germination at 28 °C for 24 h. Uniformly germinated seeds with radicle lengths of 0.5–1 cm were selected and transplanted into 96-well hydroponic boxes using Yoshida nutrient solution (Coolaber, Beijing, China) based on the conventional formulation recommended by the International Rice Research Institute [47]. The seedling cultivation was performed in three sequential stages, including an initial growth in 0.5 mM CaCl_2_ solution for 3 days, followed by transfer to half-strength N nutrient solution for 3 days, and finally in full-strength nutrient solution for 5 days. Subsequently, half of the HJ753 and DJ8 seedlings were subjected to LN treatment comprising 0.28 mM N, while the other half were maintained under NN conditions consisting of 1.40 mM N for 10 days. The nutrient solution was replaced every 24 h throughout the treatment period. After 10 days of the N treatment, root samples from both varieties under each N regime were collected for root length measurement, enzyme activity assays, and transcriptome sequencing (Figure 7).

### 4.2. Enzyme Activity Assays

The activities of NR, GS and GOGAT were determined using 0.1 g of root tissue from rice seedlings using the soil NR assay kit (BC0085), GS activity assay kit (BC0915), and GOGAT assay kit (BC0075), respectively, following the manufacturer’s (Solarbio Life Sciences, Beijing, China) instructions.

### 4.3. Determination of N Content

Root tissues from 10 rice plants were ground into a homogeneous powder. Exactly 0.2 g of the root powder was moistened with an appropriate amount of deionized water, then mixed with 5 mL of concentrated sulphuric acid and 2 g of accelerator mixture, consisting of potassium sulphate and copper sulphate in a 300 mL digestion tube. The roots were then digested at 250 °C for approximately 30 min until the sulphuric acid produced dense white fumes, after which the digestion was carried out at 400 °C until the solution became dark brown, followed by cooling at room temperature. Thereafter, the Kjeldahl N determination apparatus was preheated using prepared sodium hydroxide solution, sulphuric acid standard solution, and mixed indicator and used to determine the N content with a blank as the control.

### 4.4. Total RNA Extraction and Library Preparation

The root samples from each biological replicate were collected, rinsed with deionised water, and immediately frozen in liquid nitrogen for storage. Total RNA was extracted from the root tissues using TRIzol reagent (Invitrogen, Carlsbad, CA, USA) according to the manufacturer’s instructions. The quality and quantity of the RNA were assessed using 1% agarose gel electrophoresis and a NanoDrop 2500 spectrophotometer (Thermo Fisher Scientific, Waltham, MA, USA). RNA purity and integrity were further evaluated using an Agilent 2100 Bioanalyzer (Agilent, Santa Clara, CA, USA). The sequencing library was constructed following a standard procedure. Briefly, mRNA was enriched from total RNA using Oligo(dT) magnetic beads that selectively bind to the poly(A) tail of eukaryotic mRNAs. The purified mRNA was then fragmented into short fragments using a fragmentation buffer. These fragmented mRNAs were subsequently reverse-transcribed into first-strand cDNA using random hexamer primers. The second strand was synthesised to generate double-stranded cDNA. The double-stranded cDNA underwent end repair, followed by adenylation (A-tailing) at the 3′ ends. Sequencing adapters were then ligated to the A-tailed cDNA fragments. Finally, the library was completed through fragment size selection, PCR amplification, and purification. Their size distribution was verified with a Bioanalyzer. All qualified libraries were sequenced by Novogene Co., Ltd. (Beijing, China) on the Illumina NovaSeq x plus platform, generating between 125 and 150 bp paired-end reads for subsequent bioinformatic analysis.

### 4.5. Transcriptome Sequencing Analysis and qRT-PCR

The raw sequencing data were initially processed using the Fastp (https://github.com/OpenGene/fastp, accessed on 14 December 2025) software to perform quality control, which included the removal of adapter sequences, elimination of low-quality reads, and filtering of reads containing poly-N bases [48]. The quality of the cleaned data was assessed based on Q20, Q30 scores, and GC content, ensuring that the resulting high-quality clean reads met the stringent requirements for subsequent downstream analyses. The high-quality paired-end reads were then aligned to the reference genome of *Oryza sativa* L. ssp. *Japonica* using HISAT2 v2.2.1, for which a genome index was pre-built. The expression level of each gene was quantified using the Fragments Per Kilobase of transcript per Million mapped reads (FPKM) method, while differential expression analysis between comparative groups was conducted using DESeq2 version 1.20.0. The DEGs were identified based on absolute log_2_ fold change (|log_2_FC|) ≥ 1, with a false discovery rate (FDR) ≤ 0.01, and a nominal *p*-value < 0.05. Gene Ontology (GO) annotation and Kyoto Encyclopedia of Genes and Genomes (KEGG) pathway enrichment analysis were performed on the DEGs using the clusterProfiler package ver 3.8.1. All raw RNA-seq data have been deposited into the National Centre for Biotechnology Information (NCBI) Sequence Read Archive (SRA) under accession number PRJNA1346532 (https://www.ncbi.nlm.nih.gov/sra/PRJNA1346532, accessed on 14 December 2025). The detailed information of samples and genetic data is included in Tables S4 and S5. For qRT-PCR validation, 2 µg of the total RNA was reverse-transcribed into first-strand cDNA using the Hifair^®^ V one-step RT-gDNA digestion SuperMix for qPCR (Yeasen, Shanghai, China), following the manufacturer’s protocol. Quantitative real-time PCR was performed using specific primer sequences (Table S6), with the Hieff^®^ qPCR SYBR Green Master Mix (Yeasen) on a QuantStudio 5 Flex Real-Time PCR System (Applied Biosystems, Waltham, MA, USA), with the rice actin gene as the internal reference gene. The relative transcript abundance of the target genes was calculated using the 2^−ΔΔCt^ method.

### 4.6. Statistical Analysis

Data were presented as the mean values of at least three biological replicates. Statistical analysis was performed using one-way analysis of variance and Student’s *t*-test, with *p* < 0.05 as the significance level, using SPSS ver 25.0. Graphical representations of the data were generated using Origin software version 2021.

## 5. Conclusions

This study systematically compared the physiological and transcriptomic responses of HJ753 and DJ8 rice cultivars under LN stress. The LN stress significantly promoted root growth in both cultivars by increasing total root length and lateral root number. Moreover, HJ753 exhibited significantly higher activities of NR and GS in its roots than DJ8, suggesting its superior N assimilation capability. Transcriptome analysis of DEGs revealed common metabolic pathways involved in the response of both cultivars to LN. Comparative expression analysis of the DEGs related to tryptophan metabolism, N metabolism and utilisation, phenylpropanoid biosynthesis, and auxin signal transduction indicated genotype-specific strategies in N signal perception and transduction, N assimilation, and phenylpropanoid-mediated defence mechanisms under LN stress. In summary, these findings provide insights into the molecular basis of LN adaptation in rice and offer theoretical support for breeding cultivars with improved tolerance to low nitrogen.

## Data Availability

The raw sequencing data have been submitted to the NCBI Sequence Read Archive (SRA) under the BioProject accession number PRJNA1346532.

## Supplementary Materials

The following supporting information can be downloaded at https://www.mdpi.com/article/10.3390/plants14243836/s1: Figure S1: Principal component analysis of treatments and correlation between samples. Figure S2: Validation of 12 selected genes by qRT-PCR. Table S1: Quantification and analysis of variance data in this study. Table S2: Summary of RNA sequencing data and mapped reads to the reference genome. Table S3: Statistical table of RNA-seq data. Table S4: Gene expression and annotation in all samples. Table S5: Gene information presented in this study. Table S6: qRT-PCR primers used in this study.

## Abbreviations

The following abbreviations are used in this manuscript:

AMT	Ammonium transporter
ARF	Auxin response factor
CCR	Cinnamoyl-CoA reductase
DAO2	Dioxygenase for auxin oxidation 2
DEG	Differentially expressed gene
DEP1	Dense panicle 1
FPKM	Fragments Per Kilobase of transcript per Million mapped reads
GO	Gene Ontology
GOGAT	Glutamate synthase
GS	Glutamine synthetase
IAA	Indole acetic acid
KEGG	Kyoto Encyclopedia of Genes and Genomes
NLP	Nin-Like protein
NR	Nitrate reductase
NRT	Nitrate transporter
NUE	Nitrogen Use Efficiency
PCA	Principal component analysis
SAUR	Small auxin-up RNA
