# Supplementary material for: Comparative Physiological and Transcriptomic Characterisation of Two Japonica Rice Cultivars Under Low Nitrogen Stress
Source: Plants (Basel). 2025 Dec 16;14(24):3836. doi: 10.3390/plants14243836 (PMC12737120; doi:10.3390/plants14243836)
Supplement: Supplementary file 1 [file plants-14-03836-s001.zip › Supplementary Figures S1 and S2.pdf]

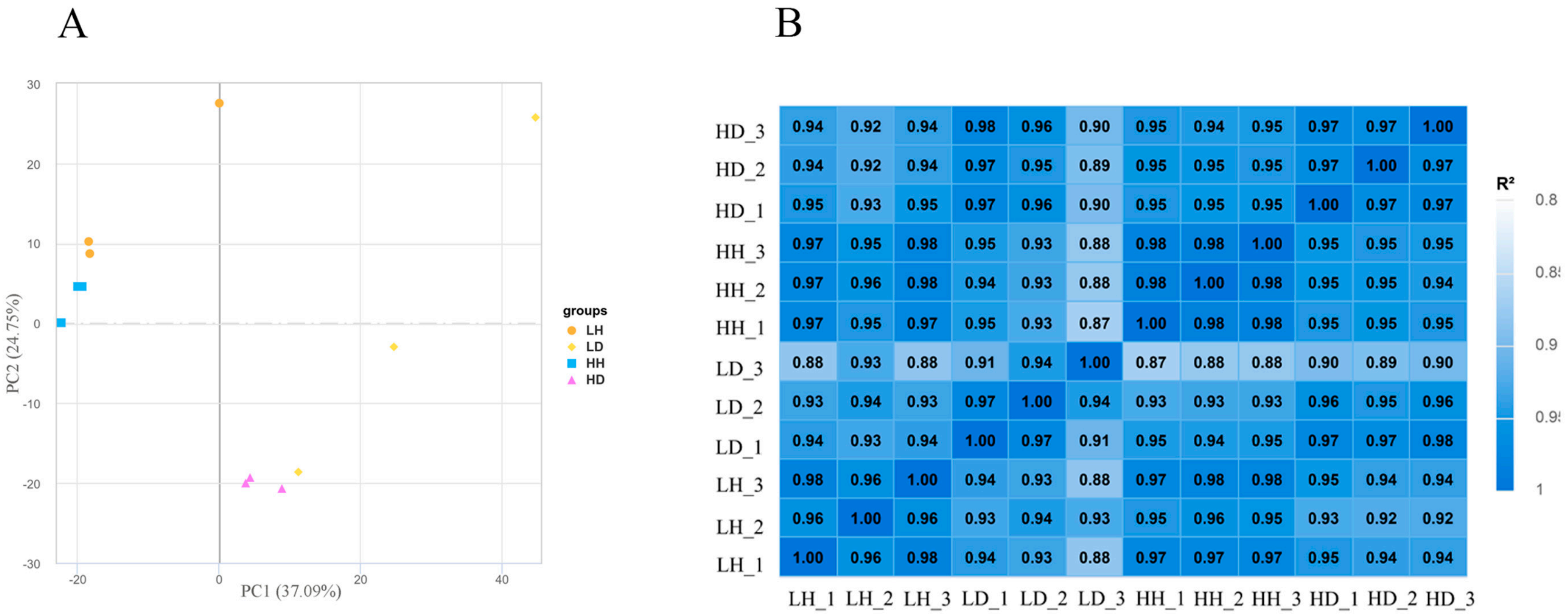

Supplementary Figure S1. Principal component analysis of treatments (A) and correlation between samples (B). The three biological performances of LH, LD, HH and HD. The LH, LD, HH and HD represent the DJ8 in low- nitrogen and normal nitrogen treatment and HJ753 in low- nitrogen and normal nitrogen treatment, respectively.

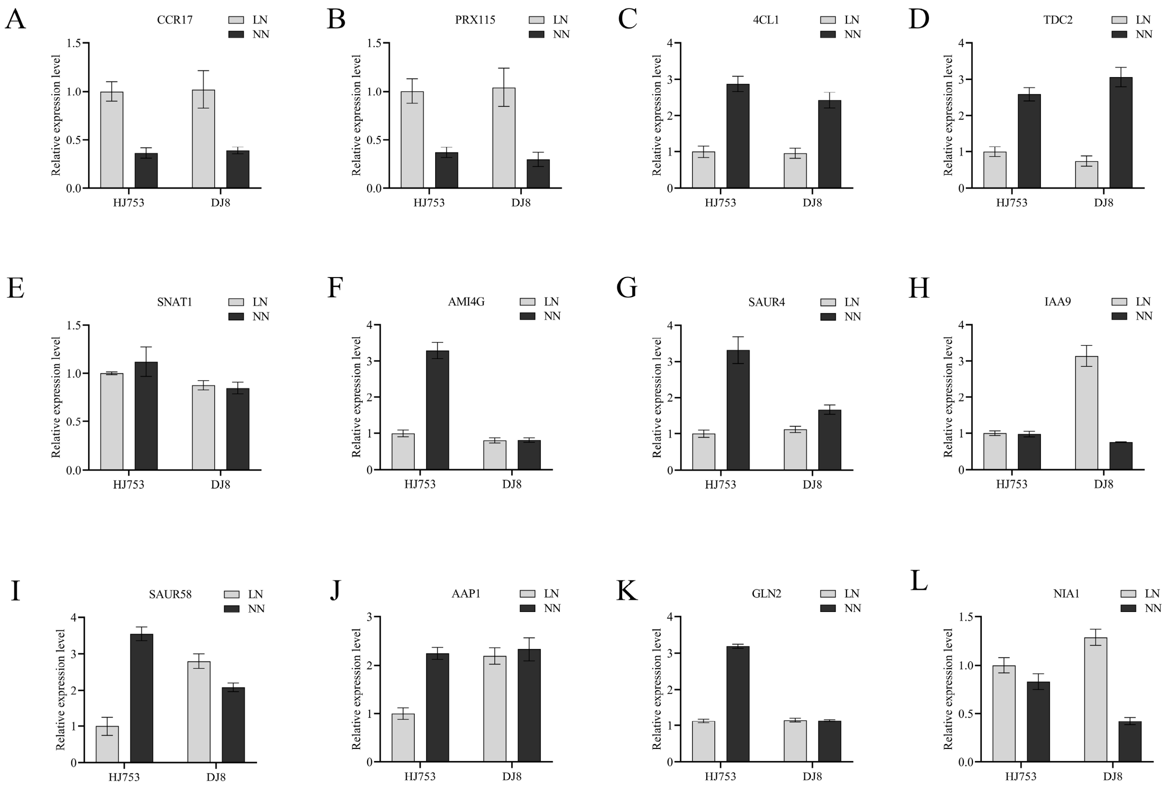

Supplementary Figure S2. (A-L)Validation of 12 selected genes by qRT-PCR. The expression levels were normalized to those of the control and are plotted as the mean ± SD.
