# Supplementary material for: Comparative Physiological and Transcriptomic Characterisation of Two Japonica Rice Cultivars Under Low Nitrogen Stress
Source: Plants (Basel). 2025 Dec 16;14(24):3836. doi: 10.3390/plants14243836 (PMC12737120; doi:10.3390/plants14243836)
Supplement: Supplementary file 1 [file plants-14-03836-s001.zip › Supplementary Table S6.pdf]

Supplementary Table S6. The primer sequences for qRT-PCR.

Genes	Primer sequence (5' to 3')	
	**Sense**	**Anti-sense**
TDC2	ACTACGACACGATAGAAGAC	GGAGATTGCCAATGAGTAAG
SNAT1	CTGGTTGCTACACTACATTC	AAGACCTTGACCCTGATATG
AMI4G	GCCAGTTCACCTGTAAGA	GCCTTCTTCTCAGTTGGA
CCR17	CGATGATCCTGAGAAGATGA	CTCTGTCCTCTTGCAGTATT
PRX115	CTTCGTCAATGGTTGTGATG	CTCAATGACATGTTGTTCGG
4CL1	GAAATCGGAGAAATACCAGTC	TCTTCTTGTAGTAGATCACCTC
SAUR4	ATGATGGGGTACTTCCGG	TAGCTCGTCGGCACCACGAAC
IAA9	GATGGTGATGGGGATTGGAT	CAGTATCTTCAGGCGTTTCA
SAUR58	GACTGCTCGTCGCCCTCCT	GAGGAGGGCGACGAGCAGTC
GLN2	CATATCAGATGCTCACTACAAG	CCTGCTCCGTTATTCTCT
AAP1	GCATACTCCTACTCCAATGT	TTCTTCATCACCTCGTTCTC
NIA1	CGACCAAGTACGGAAAGTA	ACCAGCAGTTGTTCATCA
Actin1	GACTCTGGTGATGGTGTCAGC	GGCTGGAAGAGGACCTCAGG
