# Supplementary material for: Comparative Physiological and Transcriptomic Characterisation of Two Japonica Rice Cultivars Under Low Nitrogen Stress
Source: Plants (Basel). 2025 Dec 16;14(24):3836. doi: 10.3390/plants14243836 (PMC12737120; doi:10.3390/plants14243836)
Supplement: Supplementary file 1 [file plants-14-03836-s001.zip › Supplementary Tables S1, S2 and S3.pdf]

Supplementary Table S1. Quantification and analysis of variance data in this study.

Sample	Nitrogen treatment	Root length (cm)	Number of lateral roots (per plant)	Number of adventitious roots (per plant)	Shoot height (cm)	Dry weight of roots (g)	The content nitrogen root (%)	Root NR activity (U/gFW)	Root GS activity (U/gFW)	Root GOGAT activity (U/gFW)
HJ753	LN	16.13±0.25a	237.33±12.90a	7.67±0.58a	29.31±1.06b	0.27±0.02c	2.40±0.07c	15.51±0.60b	11.98±0.43b	153.52±5.38a
	NN	13.22±0.76c	204.67±10.69b	7.00±1.00a	33.23±0.81a	0.39±0.03a	2.79±0.02a	17.66±0.21a	14.23±0.35a	162.57±7.13a
DJ8	LN	15.19±0.26b	215.33±15.53ab	7.33±0.58a	25.42±0.75c	0.21±0.03d	2.01±0.02d	12.96±0.57c	10.37±0.18c	129.51±6.12c
	NN	13.57±0.46c	193.67±9.02c	6.33±1.53a	32.47±0.70a	0.33±0.02b	2.51±0.09b	16.51±0.94ab	13.63±0.47a	152.49±6.21a

Different lowercase letters indicate significant differences (at the $P<0.05$ level) among the same variety under different nitrogen treatments.

Supplementary Table S2. Summary of RNA sequencing data and mapped reads to the reference genome (*Oryza sativa* L.)

ID	Total	Total mapped	Uniquely	Multiple	Reads map to '+'	Reads map to '-'	Reads mapped in proper
HJ753_LN_1	46364120	39529709(85.26%)	38534520(83.11%	995189(2.15%)	19252929(41.53%)	19281591(41.59%)	37112886(80.05%)
HJ753_LN_2	46247056	38921416(84.16%)	37900113(81.95%	1021303(2.21%)	18937485(40.95%)	18962628(41.0%)	36045702(77.94%)
HJ753_LN_3	38962078	33633175(86.32%)	32839909(84.29%	793266(2.04%)	16411921(42.12%)	16427988(42.16%)	32115304(82.43%)
DJ8_LN_1	51562570	41608915(80.7%)	40436010(78.42%	1172905(2.27%)	20208227(39.19%)	20227783(39.23%)	38522816(74.71%)
DJ8_LN_2	48515996	39925427(82.29%)	38866927(80.11%	1058500(2.18%)	19423966(40.04%)	19442961(40.08%)	37075386(76.42%)
DJ8_LN_3	44524702	36764274(82.57%)	35711123(80.21%	1053151(2.37%)	17850469(40.09%)	17860654(40.11%)	34167636(76.74%)
HJ753_NN_	47446394	40621367(85.62%)	39556807(83.37%	1064560(2.24%)	19763631(41.65%)	19793176(41.72%)	37662228(79.38%)
HJ753_NN_	46658146	38994262(83.57%)	38017036(81.48%	977226(2.09%)	18999781(40.72%)	19017255(40.76%)	36482682(78.19%)
HJ753_NN_	44972080	40108677(89.19%)	39135218(87.02%	973459(2.16%)	19565792(43.51%)	19569426(43.51%)	36497070(81.15%)
DJ8_NN_1	50510860	42028586(83.21%)	40942542(81.06%	1086044(2.15%)	20470917(40.53%)	20471625(40.53%)	39532798(78.27%)
DJ8_NN_2	46204538	41275631(89.33%)	40145170(86.89%	1130461(2.45%)	20063360(43.42%)	20081810(43.46%)	38376768(83.06%)
DJ8_NN_3	48071150	41388367(86.1%)	40242915(83.72%	1145452(2.38%)	20114308(41.84%)	20128607(41.87%)	38486208(80.06%)

ID, sample analysis number; Total Reads: number of Clean Reads by the single end; Mapped Reads, number of Reads mapped to the reference genome and percentage of Clean Reads; Uniquely Mapped Reads, number of Reads mapped to the unique position of the reference genome and percentage of Clean Reads; Multiple Map Reads, number of Reads mapped to the multiple positions of the reference genome and percentage of Clean Reads; Reads Map to "+" described the number of reads to the forward strand of the reference genome and the percentage of Clean Reads; Reads Map to "−" described the number of reads to the reverse strand of the reference genome and the percentage of Clean Reads.

The HJ753_LN, DJ8_LN, HJ753_NN and DJ8_NN respectively represent HJ753 and DJ8 under low nitrogen treatment, and HJ753 and DJ8 under normal nitrogen treatment.

Supplementary Table S3. Statistical table of RNA-seq data.

ID	Raw Reads	Raw Bases	Clean Reads	Clean Bases	Error Rate	Q20	Q30	GC
HJ753_LN_1	47.57M	7.13G	46.36M	6.95G	0.01	98.38%	95.43%	50.20%
HJ753_LN_2	47.51M	7.13G	46.25M	6.94G	0.01	98.27%	95.08%	49.07%
HJ753_LN_3	39.52M	5.93G	38.96M	5.84G	0.01	99.29%	97.15%	49.09%
DJ8_LN_1	53.32M	8.00G	51.56M	7.73G	0.01	98.30%	95.17%	49.81%
DJ8_LN_2	49.84M	7.48G	48.52M	7.28G	0.01	98.36%	95.43%	49.96%
DJ8_LN_3	45.88M	6.88G	44.52M	6.68G	0.01	98.32%	95.22%	50.13%
HJ753_NN_1	48.84M	7.33G	47.45M	7.12G	0.01	98.27%	95.10%	50.28%
HJ753_NN_2	47.54M	7.13G	46.66M	7.00G	0.01	99.11%	97.36%	48.88%
HJ753_NN_3	46.26M	6.94G	44.97M	6.75G	0.01	99.00%	97.05%	50.37%
DJ8_NN_1	51.05M	7.66G	50.51M	7.58G	0.01	99.02%	97.18%	49.64%
DJ8_NN_2	47.66M	7.15G	46.20M	6.93G	0.01	98.35%	95.34%	50.09%
DJ8_NN_3	49.87M	7.48G	48.07M	7.21G	0.01	98.49%	95.67%	50.07%

ID, ample analysis number; Raw Reads: Reads in the original data; Raw Bases: The number of bases in the original data; Clean Reads: Clean data; Clean Bases: The number of bases in the clean data; Error Rate: The overall sequencing error rate of the data; Q20: The percentage of bases with a Phred value greater than 20 among the total bases; Q30: The percentage of bases with a Phred value greater than 30 among the total bases; GC: The percentage of G and C among the four bases in the clean data.

The HJ753_LN, DJ8_LN, HJ753_NN and DJ8_NN respectively represent HJ753 and DJ8 under low nitrogen treatment, and HJ753 and DJ8 under normal nitrogen treatment.
